# Confidence as a diagnostic tool for perceptual aftereffects

**DOI:** 10.1038/s41598-019-43170-1

**Published:** 2019-05-09

**Authors:** Regan M. Gallagher, Thomas Suddendorf, Derek H. Arnold

**Affiliations:** School of Psychology, The University of Queensland, Brisbane, Australia

**Keywords:** Perception, Sensory processing

## Abstract

Perceptual judgements are, by nature, a product both of sensation and the cognitive processes responsible for interpreting and reporting subjective experiences. Changed perceptual judgements may thus result from changes in how the world appears (perception), or subsequent interpretation (judgement). This ambiguity has led to persistent debates about how to interpret changes in decision-making, and if higher-order cognitions can change how the world looks, or sounds, or feels. Here we introduce an approach that can help resolve these ambiguities. In three motion-direction experiments, we measured perceptual judgements and subjective confidence. We show that each measure is sensitive to sensory information and can index sensory adaptation. Each measure is also sensitive to decision biases, but response bias impacts the central tendency of decision and confidence distributions differently. Our findings show that subjective confidence, when measured in addition to perceptual decisions, can supply important diagnostic information about the cause of aftereffects.

## Introduction

A central challenge in perception research is to understand how the world looks, feels, and sounds, as opposed to how it is remembered, imagined, or judged. But perceptual judgements are, by nature, a product of both sensation and the cognitive operations responsible for producing measurable behaviour. Changes in perceptual decision-making could thus equally arise from changes to sensory encoding and perception, or from decisional processes that operate independently of perception. This ambiguity has fostered persistent debate regarding the degree to which our cognitions — like imaginations, motivations, or beliefs — can change the sensory processes that determine what we perceive^1–5^. Here we introduce a new approach that can help shed light on whether or not changes in perceptual judgements reflect changes in perception.

Much of what we know about human perception has resulted from investigating sensory aftereffects^6–8^. An aftereffect is a change in the measured boundary between perceptual categories, which can result from prolonged and repeated exposures to a specific stimulus^9–12^, or rapidly from a single brief exposure to a test stimulus^13–17^. It remains a matter of debate, however, how best to dissociate perceptual from non-perceptual effects on decision-making^1,3,18,19^. We examine this problem by using motion-direction judgements and subjective confidence, and we show that reports of high and low confidence provide important information about the cause of changes in perceptual decisions.

Perceptual decisions are often measured by forced-choice categorisations. Participants might be tasked with determining which binary category a test stimulus belongs, for example motion direction (left or right), orientation (clockwise or counter-clockwise tilt), or facial characteristics (masculine or feminine). Ambiguous stimuli (e.g. incoherent motion, near vertical orientations, or androgynous faces) represent the boundary between these categories and, in an unbiased observer, are equally likely to be categorised as belonging to either category.

Metacognition research shows that individuals can accurately predict their own ability to discriminate between perceptual categories^20–25^. Decisions likely to be correct elicit greater feelings of confidence, whereas decisions likely to be incorrect (or made by guessing) carry lower confidence. An individual’s boundary between categories is their point of subjective equality (PSE), characterised by both probabilistic responding and subjective uncertainty.

Since typical observers can accurately rate their own performance in perceptual decision tasks, confidence might provide important information about whether an aftereffect represents a change in a perceptual or decision boundary. On one hand, aftereffects caused by changes to sensory encoding should equally impact categorical decisions and confidence reports, because the sensory evidence underlying both judgements has changed. On the other hand, aftereffects arising only from changes to decision processes^18,26^ might selectively or disproportionately impact decisions made under uncertainty, without corresponding changes in confidence.

**Figure 1:**
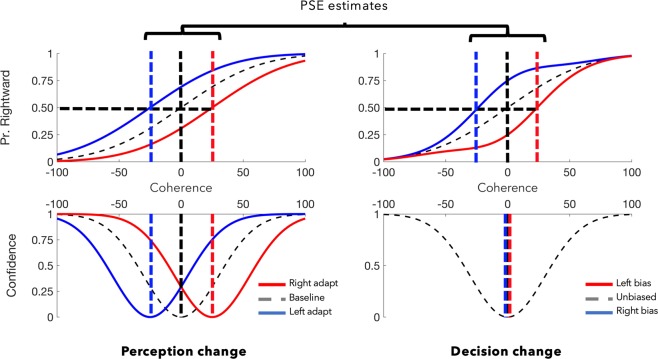
Confidence can distinguish perceptual from non-perceptual effects on decision-making. Here we illustrate hypothetical data sets for motion-direction judgments. Categorical decisions (“are stimulus elements predominantly moving left, or right?”) are plotted above as a function of motion coherence (the proportion of elements physically moving left or right). Expressions of confidence (“how confident are you in your decision?”), as a function of motion coherence, are plotted below. Both measures are assumed to scale with the strength of sensory evidence. On the left we depict patterns of results expected from changed sensory evidence; if sensory evidence changes, previously ambiguous inputs should *look* as if they are moving right (blue line) or left (red). Categorical decisions and confidence are expected to shift in tandem as both judgments are informed by sensory evidence. On the right we depict expected responses from people making different decisions when uncertain; if decisions change because people make different decisions regarding ambiguous inputs, categorical decisions might shift primarily for stimuli that are associated with low confidence. In this case, categorisation changes are proportional to (lack of) confidence, but the distribution of confidence remains unchanged (see Supplement 1 for Matlab code).

Figure 1 depicts anticipated patterns of responding when decisions are biased by changes to sensory evidence (left), and when people make biased decisions about ambiguous inputs (right). To depict the impact of sensory adaptation on decision-making, the unbiased decision and confidence functions have been laterally shifted. The lateral shift reflects a change in central tendency of categorical decision-making on the basis of updated encoding of motion direction. To depict the impact of non-perceptual decision biases, an unbiased decision function has been multiplied by a bias factor that scales with an unchanged region of uncertainty, which results in non-symmetrical functions (with a slight increase in slope about the point of maximal uncertainty). The biased decision functions are largely similar to response patterns expected from changes to sensory evidence, but a clear dissociation is depicted in the confidence results.

It might seem odd to suggest that people could make different perceptual decisions even if perception is unchanged. This is possible, however, if people are implicitly or explicitly encouraged to make different decisions about ambiguous information. An instruction to dwell on immoral actions, for instance, could implicitly encourage people to regard ambient lighting as ‘darker’ than if they dwelt on moral actions^4,27^. Or people might have a tendency to repeat decisions that were effective in the past, which could result in ambiguous inputs being judged as more similar to previous inputs^28–30^. Either scenario could result in people making different decisions about ambiguous information, even if perception is unchanged. Crucially, as neither scenario involves changes to perception, the range of inputs associated with low confidence should also be unchanged. If this is true, confidence could be used to resolve ambiguity about the underlying cause of many aftereffects. To test this, we measured decisions and associated confidence in three motion-direction experiments.

In Experiment 1 we examined the motion-direction aftereffect, where decision changes are known to reflect changes to physiological processes^31–33^. As predicted, we find that categorical direction decisions and confidence measures are equally impacted by adaptation. In Experiment 2 we instruct participants to default to biased decision strategies when they encounter ambiguous tests. This experiment demonstrates that categorical decisions can change without any change to perception, and when this happens, expressions of confidence remain unchanged. Finally, in Experiment 3, we examine serial dependency (the impact of prior stimuli on subsequent judgments). Results suggest that the most recent stimulus (1-back) changes perception, impacting categorical decisions and confidence equally. The stimulus before last (2-back), however, seems to *selectively* impact categorical judgements, consistent with a post-perceptual influence on decisions. Overall, our data show that confidence reports can provide diagnostic evidence for determining whether or not changes in decision-making reflect changes in perception.

## Method

### Participants

All participants were recruited from the University of Queensland’s Psychology department, and were naïve as to the purpose of the experiments. Experiment 1 had a sample size of 15, which provides very high power to detect perceptual adaptation effects (these are typically large; Cohen’s d > 0.8). This conservative sample size estimate was chosen because the effect of adaptation on confidence was yet unknown. Experiment 2 also had a sample size of 15 to match Experiment 1. The sample size for Experiment 3 was increased to 24 because serial dependence effects are smaller than adaptation effects (although still moderately large, Cohen’s d > 0.5).

Two participants were excluded from Experiment 3 because either their category judgements (n = 2) or confidence judgements (n = 1) could not be adequately modelled using a standard psychometric function fit, so the final sample size of Experiment 3 was 22. Participants in Experiment 2 were postgraduate Psychology and UQ Perception Lab members, all other participants were recruited from a first-year student pool.

### Ethics

Ethical approval for all experiments was obtained from the University of Queensland’s (UQ) Ethics Committee, and all experimental tasks were performed in accordance with the UQ guidelines and regulations for research involving human participants. Each participant provided informed written consent to participate in the study and were made aware that they could withdraw at any moment from the study without prejudice or penalty. First year students received course credit in exchange for participation.

### Stimuli and apparatus

In all tasks the motion stimuli were generated using Matlab software and the Psychophysics Toolbox^34,35^. Stimuli were presented on Dell P791 monitors (1024 × 768 pixels) with a refresh rate of 60 Hz. The dot-motion stimuli were single-pixel dots rendered blue (RGB = 0,0,255). Coherent motion signals were created by translating dots in the coherent direction by 1 pixel on each frame. To avoid participants tracking individual dots, a new subset of dots (equal to the % dot coherence) were selected at random on each frame. All other dots were moved to a new and randomly selected location on each frame, within the aperture, to create a background of incoherent motion.

Test stimuli in all experiments consisted of 100 blue dots, shown against a grey background. Dot coherence values ranged from −30 (30% leftward) through 0 (random motion) to +30 (30% rightward). Test stimuli in Experiments 1 and 2 were set to one of 11 coherence values [−30 −20 −10 −6 −3 0 +3 +6 +10 +20 +30], presented in a random order. In Experiment 3, results were analysed on the basis of the physical direction of the previous stimulus. We therefore excluded the 0% coherence stimulus and test stimuli were set to one of eight coherence values [−30 −15 –5 −1 +1 +5 +15 +30].

In Experiment 1, adapting stimuli consisted of 100 dot stimuli, each 1 pixel in size. A coherent motion signal was achieved by selecting 30 dots at random on each frame to be displaced left or right by one pixel. All other dots were redrawn at random locations. Again, to avoid participants tracking individual dots, a new subset of dots (equal to the % dot coherence) were selected at random on each frame. All other dots were moved to a new and randomly selected location on each frame, within the aperture, to create a background of incoherent motion. Adapting motion was in one direction for the first half of the block of trials, and in the other direction for the second half of the block, with the initial adaptation direction determined at random for each participant.

**Figure 2:**
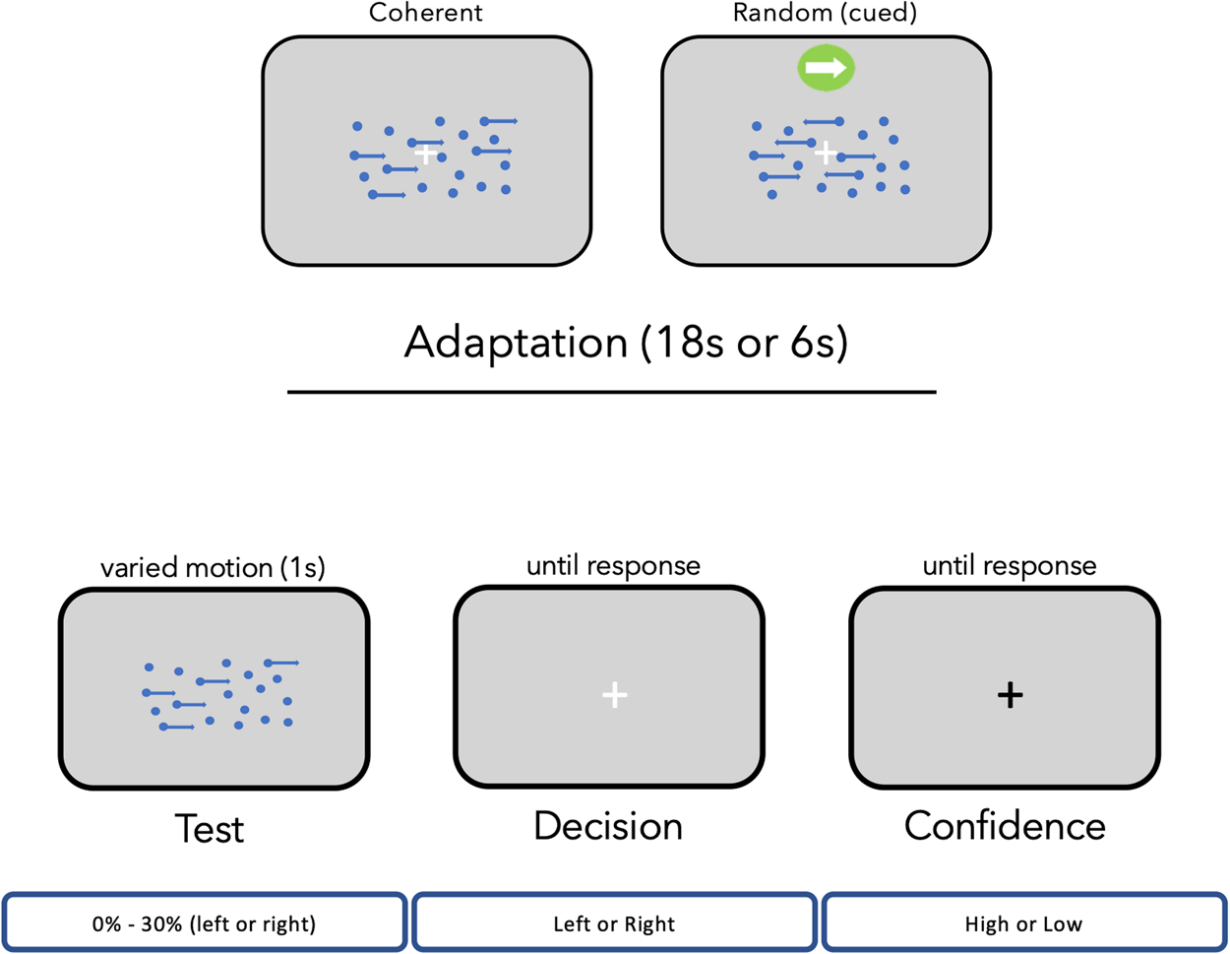
Procedures for each experiment. Participants adapted to stimuli that depicted either coherent motion or random motion and a static direction cue. Each trial within a testing block consisted of an adaptation phase (except for baseline) followed by a dot test probe. The direction of the adapting stimulus (left or right) was consistent within the first half of a block, and then changed direction for the second half. Adapting stimuli appeared for 18 s on the first trial of each block, and on the middle trial when the stimulus changed direction, and for 6 s on all other trials. Dot test probes were present for 1 s, appearing on the second frame after the adapting stimulus disappeared. A new trial began once participants had recorded their direction decision (left or right) and reported their confidence (whether they had confidence in their decision — yes/high confidence or no/low confidence).

In Experiment 2 ‘adapting’ stimuli were similar to Experiment 1, with two exceptions. First, motion coherence was 0, so *all* dots were redrawn at random locations on each frame. Second, a static direction cue was presented directly above the stimulus window — an arrow pointing to the left or right (see Fig. 2). The static direction cue was in one consistent direction for the first half of a block of trials, and reversed for the second half of the testing block. The initial cue direction was determined at random for each participant.

In Experiments 1 and 2, each test stimulus was presented 10 times, and repeated for each adaptation or cue direction (totalling 220 stimulus observations). There were no adapting stimuli in Experiment 3, so each test stimulus was viewed 55 times (totalling 440 stimulus observations).

### Procedure

Participants received a written instruction of the experimental procedure. After reading the instructions, participants were then verbally queried whether they understood how to report their responses. All understood the direction judgement was to probe which direction the test appeared to move (left or right), and how to respond if they were unsure (which was varied according to experiments). Likewise, all participants understood to report whether they had high or low confidence in each response or, if it was more intuitive, to report whether they thought their guess was likely correct or incorrect. All participants verbally acknowledged that they understood both the direction and the confidence responses.

When participants had acknowledged they understood the instructions, they then sat comfortably in front of the monitor at a distance of approximately 55 cm, rested their fingers on the keyboard’s arrow buttons, and fixated a central cross-hair. If there was an adaptation phase, the adapting stimulus was presented for 18 seconds on the first trial in each block, and again on the middle trial (when adapting motion direction reversed). On all other trials the adapting stimulus lasted for six seconds.

On each test trial, participants were presented with one of the 11 tests for 1 second. After the stimulus presentation period concluded, participants could immediately indicate whether the test had appeared to be moving left (by pressing the left arrow key) or right (by pressing the right arrow key). In Experiments 1 and 3, participants were instructed to provide their best guess if they could not determine which direction the test was moving. In the adaptation (but not baseline) phase of Experiment 2, participants were instructed to default their response to the direction congruent (Experiment 2a) or incongruent (Experiment 2b) with the static reference cue presented on that trial. Participants in Experiment 2 completed both tasks (congruent and incongruent) in a random and counterbalanced order.

Once participants had completed a direction judgment, the white fixation cross hair turned black, which prompted the participant to make a confidence judgment. Participants could immediately report whether they felt confidence in their response (by pressing the up arrow – a high confidence response) or not (by pressing the down arrow – a low confidence response). The fixation cross turned white once the confidence response had been recorded, and a new trial started after a 50 ms delay.

## Experiment 1

We first checked that categorical boundaries and perceptual aftereffects can be equally estimated from categorical decisions and confidence judgements. People made categorical direction decisions (left/right) and confidence judgments (low/high) about tests that varied in direction and motion coherence. Cumulative Gaussian functions were fit to each participant’s distribution of rightward direction judgements as a function of motion coherence (see Methods). The 50% points were taken as estimates of the point of subjective equality (PSE) — the stimulus value equally likely to be judged as moving left or right.

A raised Gaussian function was fit to each participant’s distribution of low-confidence responses, and the peak of the fitted function was taken as a second PSE estimate — the point of peak uncertainty. All *t*-tests reported are two-tailed repeated measures tests for equality of means, unless stated otherwise. All Bayes’ factors were estimated using JASP software^36^, with the default Cauchy prior width of 0.707.

### Baseline

**Figure 3:**
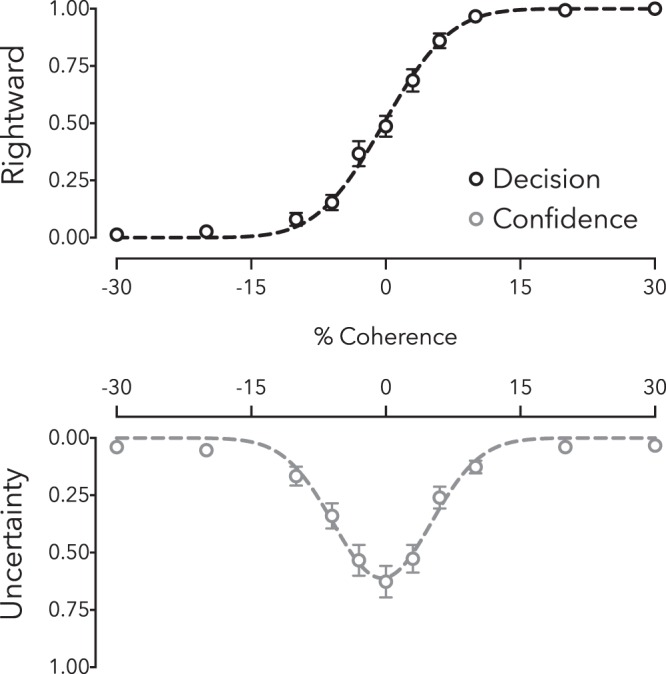
Baseline data (N = 15). Distributions of reported motion direction (left or right) and confidence (high or low) as a function of dot coherence (percent) and direction (negative values left, positive right). Data and best-fit functions are depicted for baseline motion-direction judgments in Experiment 1. The inflection point of the function fitted to decision data (blue), and the peak of the function fit to confidence data (red) each estimate the point of subjective equality. Depicted data are averaged across all participants, but parameter estimates are derived from individual fits. Error bars depict ± 1 SEM.

To illustrate our approach, in Fig. 3 we depict distributions of categorical decisions (top) and expressions of uncertainty (or low-confidence; bottom). As can be seen, functions fit to these distributions can provide a closely matched PSE estimate (direction decisions, M = −0.43, SD = 1.60; confidence reports, M = −0.51, SD = 1.03; difference *t*_14_ = 0.18, *p* = 0.862, BF_10_ = 0.27). A comparison of the standard deviation of the two psychometric functions also indicates close agreement in measurement precision (decision, M = 6.32, SD = 4.31; confidence, M = 5.37, SD = 1.90; difference *t*_14_ = 0.84, *p* = 0.416, BF_10_ = 0.36). These baseline data in Fig. 3 are averaged across all participants, but PSE and sensitivity estimates were derived from individual function fits.

We examined decision and confidence further to explore whether spontaneous biases in baseline PSE would co-vary with uncertainty. Participants were first split according to whether their decisional PSE estimate at baseline was negative (M = −1.80, SD = 1.15) or positive (M = 0.78, SD = 0.66). As one might anticipate, this resulted in two groups with different decisional PSEs (independent *t*_13_ = 5.42, *p* < 0.001). Cohen’s d effect size estimates and Bayes Factor analyses show a very large effect of splitting participants according to the direction of their spontaneous decision PSE at baseline (decision effect d = −2.81 95%CI 1.31–4.25, BF_10_ = 163.45).

**Figure 4:**
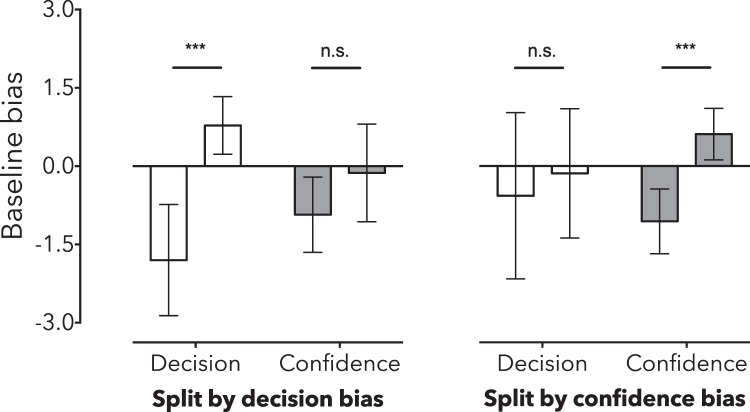
Split of baseline data according to whether (left) the point of subjective equality (PSE) and (right) peak uncertainty estimates were negative or positive. Error bars depict 95% CIs.

Grouping people based on the sign of their baseline decision bias had no clear impact on confidence measures (positive M = −0.94, SD = 0.78, negative M = −0.13, SD = 1.12; independent *t*_13_ = 1.60, *p* = 0.136). These data are ambiguous, however, as a Bayes Factor analysis favoured neither the null or the alternate hypothesis (BF_10_ = 0.98; see Fig. 4).

Baseline data were then split according to participants’ confidence biases. Confidence data were split according to whether peak uncertainty at baseline was associated with a leftward (M = −1.07, SD = 0.67) or rightward (M = 0.61, SD = 0.60) motion test value. This resulted in a large difference in peak uncertainties (independent *t*_13_ = 4.74, *p* < 0.001). Cohen’s d effect size estimates and a Bayes Factor analysis showed a very large effect of splitting participants according to the direction of their peak uncertainty at baseline (confidence effect d = −2.60 95%CI 1.11–4.03, BF_10_ = 59.51). Categorical decision data was also split into groups based on baseline confidence, but these groups did not differ in baseline categorical decision PSEs (positive M = −0.14 SD = 1.48, negative M = −0.57 SD = 1.72; independent *t*_13_ = 0.47, *p* = 0.646). A Bayes Factor analysis showed that this effect slightly favoured the null hypothesis (BF_10_ = 0.49).

### Coherent motion adaptation

In the adaptation phase, participants adapted to a dot motion stimulus with 30% coherence, moving either to the left or right (see Methods for further details). Adaptation data for each participant produced two decision distributions; one distribution depicts responses for left-adapted trials, the other for right-adapted trials (each indicates the proportion of stimuli judged as having moved predominantly rightward as a function of test dot coherence and direction). The impact of adaptation was estimated as the difference between these two PSE estimates for each individual. Adaptation data for each participant also produced two distributions of confidence, indicating the proportion of low-confidence responses as a function of test dot coherence and direction (again for left-adapted and right-adapted trials).

**Figure 5:**
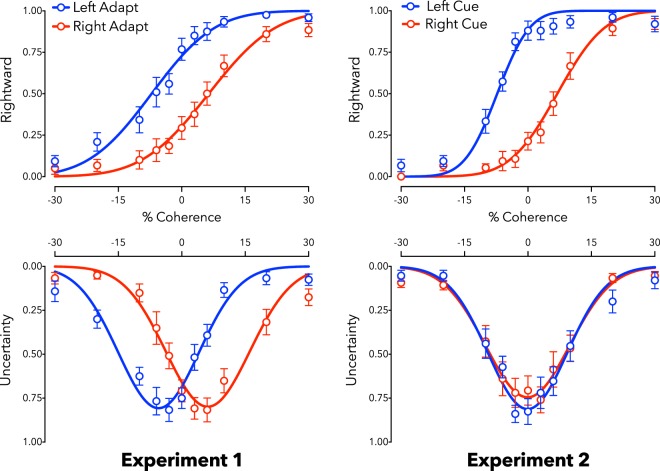
Reported motion direction (left or right, top row) and low-confidence (uncertainty, bottom row) as a function of dot coherence (percent) and direction (negative values left, positive right). Data are shown along with best-fit functions for adaptation data in Experiments 1 (left column) and 2 (right column). Both Experiments are associated with changed direction decisions (see top row), but only Experiment 2 is associated with shifts in confidence (bottom row; see main text for further details). Depicted data are averaged across participants, but statistical tests were based on individual data fits. N = 15; Error bars depict ± 1 SEM.

Adaptation to coherent motion had a robust impact on direction decisions, with left adaptation (L_PSE_ = −11.67, SD = 7.31) and right adaptation (R_PSE_ = 11.22, SD = 6.76) producing different PSE estimates (*t*_14_ = 6.90, *p* < 0.001). These data are depicted in Fig. 5 (left panel, top). The same pattern of results was observed for confidence data. Adaptation robustly impacted measures of uncertainty, with left adaptation (L_CONF_ = −10.02, SD = 5.79) and right adaptation (R_CONF_ = 9.93, SD = 5.27) producing different confidence profiles (*t*_14_ = 7.98, *p* < 0.001). The effect of adaptation on decision data (ΔPSE = 22.89, SD = 12.84) and confidence data (ΔCONF = 19.95, SD = 9.69) was not statistically different (*t*_14_ = 1.73, *p* = 0.106). These data are also depicted in Fig. 5 (left panel, bottom).

The results of null hypothesis statistical tests are also supported by Bayes Factor analyses, with strong evidence favouring adaptation-induced changes (decision BF_10_ = 2.92 × 10^3^; confidence BF_10_ = 1.28 × 10^4^). Cohen’s *d* effect size estimates for each measure show that adaptation produces large aftereffects (decisions *d* = 1.78, 95% CI 0.94–2.60; confidence *d* = 2.06, 95% CI 1.14–2.96). It is important to note that decision and confidence measures provide a statistically equivalent measure of the aftereffect (note the overlapping CIs). This is important as it argues against future dissociations arising because confidence measures provide a less sensitive measure of adaptation. The two measures have provided an equivalent estimate of the motion aftereffect, which is known to result from physiological changes that impact perception^31,32^.

## Experiment 2

Experiment 1 showed that the motion aftereffect can be measured equally well using categorical perceptual decisions or confidence judgments. In Experiment 2 we assess the logical counterpoint: how will an aftereffect that results from people making different decisions about ambiguous inputs, without any perceptual changes, impact perceptual decisions and measures of confidence? To achieve this, in Experiment 2 people ‘adapt’ to motion with no coherent direction. Participants are then instructed to make a default decision (either left or right, according to instruction) when they encounter subjectively ambiguous tests. This experiment is conceptually similar to a previous study^26^, where participants were instructed to adopt a biased pattern of response under conditions of uncertainty. Our experiment will complement this study, by also examining the impact of this instruction on a measure of confidence.

### Adapt to random motion

Participants ‘adapted’ to a random dot motion stimulus (0% coherence), which should have no systematic impact on sensory encoding of motion direction^32^. We also instructed participants to adopt a default decision, rather than guess, if they were unable to determine the direction of a test. The important question is the degree to which decision and confidence judgments are dissociable by systematically biased categorical decisions about ambiguous inputs.

A static direction cue (an arrow pointing to the left or right) was presented above the ‘adaptor’ (see Methods for further details). The arrow either pointed to the left or right, and we prompted people to report either the direction consistent (congruent condition) or inconsistent (incongruent condition) with the arrow when test motion direction was ambiguous. Some research has suggested that attractive biases and contrastive biases represent the opposite effects of decision and perception on perceptual judgements respectively^19^. We therefore used both a congruent condition and an incongruent condition to show that decision biases could manifest equally as assimilative or repulsive aftereffects.

The congruent condition is expected to produce category decisions consistent with an assimilative aftereffect (when tests are judged as being *similar* to an ‘adaptor’)^37^. The incongruent condition is expected to produce categorical decision changes consistent with a negative aftereffect (when tests are judged as being *dissimilar* to an ‘adaptor’, as in the classic motion-direction aftereffect measured in Experiment 1).

### Categorical direction decisions

When faced with an ambiguous test, participants were more likely to report that the test stimulus was moving in the same direction as the cue in the congruent task, and this bias impacted PSE estimates (L_PSE_ = 8.21, SD = 6.58; R_PSE_ = −7.15, SD = 3.48; difference, *t*_14_ = 7.50, *p* < 0.001). Participants were less likely to report the same direction in the incongruent task, which also impacted PSE estimates (L_PSE_ = −8.81, SD = 4.44; R_PSE_ = 7.77, SD = 4.40; difference, *t*_14_ = 8.33, *p* < 0.001; see Fig. 5). These were large effects (congruent *d* = 1.93, 95% CI 1.05–2.79; incongruent *d* = 2.15, 95% CI 1.20–3.08).

Bayes Factor analyses favoured the hypotheses that decision biases can produce data consistent with both assimilative (congruent BF_10_ = 6.62 × 10^3^) and contrastive (incongruent BF_10_ = 2.01 × 10^4^) aftereffects (see Fig. 5). A comparison of slope estimates showed no significant difference in participants ability to maintain a stable criterion across conditions. The standard deviation of the psychometric functions did not differ in the congruent condition (L_SD_ = 9.54, R_SD_ = 7.41, p = 0.170), nor did they differ in the incongruent condition (L_SD_ = 7.60, R_SD_ = 9.03, p = 0.381).

### Confidence results

When faced with an ambiguous test, participants were no more likely to report low (or high) confidence for different tests in either the congruent (L_CONF_ = −0.12, SD = 1.13; R_CONF_ = 0.62, SD = 1.07; difference, *t*_14_ = 1.15, *p* = 0.268) or incongruent (L_CONF_ = 0.02, SD = 1.26; R_CONF_ = −0.38, SD = 1.02; difference, *t*_14_ = 0.85, *p* = 0.410; see Fig. 5) tasks.

### Dissociation of decision and confidence effects

There was a clear dissociation between PSE estimates from direction decisions and confidence judgments in the congruent (decision ΔPSE = 13.46, SD = 6.20; confidence ΔCONF = 0.50, SD = 1.68; difference, *t*_14_ = 9.23, *p* < 0.001) and incongruent (decision ΔPSE = 14.94, SD = 7.91; confidence ΔCONF = 0.41, SD = 1.85; difference, *t*_14_ = 7.07, *p* < 0.001) tasks. Effect size estimates for these differences were large (congruent *d* = 2.01, 95% CI 1.11–2.89; incongruent *d* = 1.62, 95% CI 0.83–2.39). The dissociation is supported by Bayes Factor analyses, which provide strong evidence for the alternate hypothesis, that categorical decision and confidence measures differed (congruent BF_10_ = 6.14 × 10^4^; incongruent BF_10_ = 3.71 × 10^3^).

In combination, Experiments 1 & 2 show that categorical decisions and confidence judgements can equally measure an aftereffect known to result from physiological processes that change perception (Experiment 1), but provide dissociable measures when ‘aftereffects’ instead result from people making different decisions about ambiguous inputs (Experiment 2). Having validated that both reports can index sensory adaptation, and also dissociate when biased decisions are made under uncertainty, we adopted it to assess the likely cause of serial dependence.

## Experiment 3

Recent studies have established a contingency between perceptual decisions and preceding tests, without any need for protracted adaptation periods^13,14^. One interpretation is that serial dependence results from rapid sensory adaptation, which measurably impacts perception on a trial by trial basis. Another interpretation suggests that serial dependence can result from post-perceptual aspects of decision-making, like working memory^19^, or from people repeating their previous response when subsequent stimuli are ambiguous^28^. We assessed these proposals by measuring serial dependencies between sequential categorical decisions and confidence judgments.

### N-back effects

**Figure 6:**
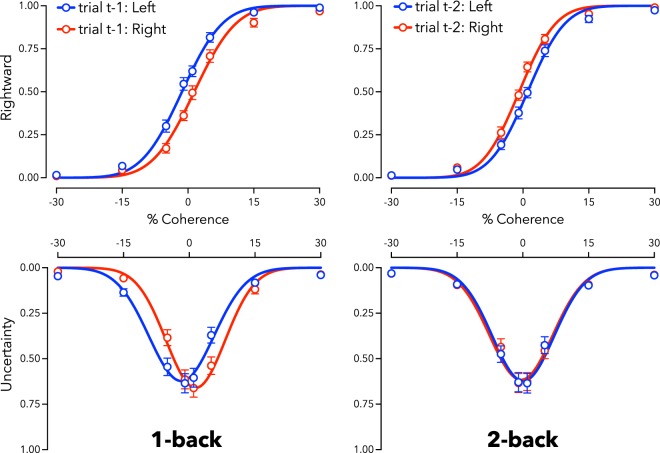
Proportion of rightward motion reported (top row) and low-confidence (uncertainty, bottom row) as a function of dot coherence (percent) and direction (negative values left, positive right). Data are divided according to the physical direction of the last test (Left panel: t-1 data), and according to the physical direction of the test two trials ago (Right panel: t-2 data). Blue data indicate that the prior trial was moving left, red that the prior trial was moving right. Depicted data are averaged across participants, but statistical tests were based on individual fits. N = 22; Error bars depict ± 1 SEM.

People made categorical direction decisions (left/right) and confidence judgments (low/high) about tests that varied in direction and motion coherence, akin to baseline trials in Experiment 1 (see Methods for further details). Trial responses were subdivided according to the direction of the last test (1-back data), and according to the direction of the test two trials prior (2-back data). Functions were fit to these data to quantify 1-back and 2-back effects on categorical decision-making, as a function of motion direction and coherence on the present test (see Fig. 6).

### 1-back effects

Participants tended to categorise tests as moving in the *opposite* direction relative to the last test (L_PSE_ = −1.50, SD = 2.38; R_PSE_ = 1.72, SD = 3.18; difference, *t*_21_ = −4.46, *p* < 0.001; see Fig. 6, left panel, top). The last trial also impacted confidence judgments. Peak uncertainty shifted in tandem with direction judgments, in the *opposite* direction relative to the last test (L_CONF_ = −1.99, SD = 1.74; R_CONF_ = 1.35, SD = 1.64; difference, *t*_21_ = −7.46, *p* < 0.001).

1-back effects can be described as large, both for categorical decisions (decision *d* = 0.95, 95% CI 0.44–1.45) and confidence (*d* = 1.59, 95% CI 0.95–2.22). Again, it is important to note that confidence provided a statistically equivalent measure of this serial dependency relative to categorical decisions (note the overlapping CIs). Bayes Factor analyses provided strong support for the hypothesis that the last trial impacted direction decisions (decision BF_10_ = 1.39 × 10^2^) and confidence judgments (BF_10_ = 6.52 × 10^4^). These effects were statistically equivalent (decision ΔPSE = 3.23, SD = 3.39; confidence ΔCONF = 3.34, SD = 2.10; difference, *t*_21_ = 0.20, *p* = 0.841), and a Bayes Factor analysis provided moderate evidence for the equivalence of the two 1-back effects (decision vs confidence BF_10_ = 0.23). This pattern of results is consistent with those obtained for the classic motion-direction aftereffect in Experiment 1, suggesting that serial dependence effects can be driven by processes that impact perception.

### 2-back effects

Participants tended to categorise tests as moving in the *same* direction as the test two trials prior (t-2; L_PSE_ = 1.34, SD = 2.98; R_PSE_ = −1.20, SD = 2.45; difference, *t*_21_ = 3.44, *p* = 0.002; see Fig. 6). There was, however, no discernible impact of this trial on confidence judgements (L_CONF_ = 0.09, SD = 1.37; R_CONF_ = −0.48, SD = 1.87; difference, *t*_21_ = 1.45, *p* = 0.161). There was also a significant dissociation between measures of aftereffect estimates (categorical decision ΔPSE = 2.54, SD = 3.46; confidence ΔCONF = 0.58, SD = 1.87; difference, *t*_21_ = 2.81, *p* = 0.011). These data are consistent with the pattern of results in Experiment 2, where an ‘aftereffect’ resulted from people making different decisions about ambiguous inputs, rather than from sensory encoding changes.

**Figure 7:**
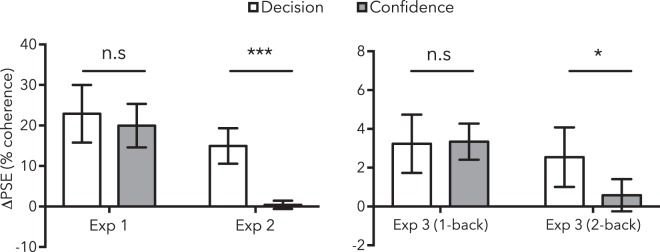
Bar graphs summarising the results of all experiments. Data depict mean (absolute) PSE changes calculated from categorical decisions (white bars) and confidence judgments (grey bars). Error bars depict 95% CIs.

The effect size estimate for 2-back categorical decision effects was moderately large, as was the effect size for differences between the decision and confidence judgments (decision *d* = 0.73 95% CI 0.26–1.20; decision/confidence difference *d* = 0.60 95% CI 0.14–1.05). Bayes factor analyses provided moderate to strong evidence in favour of both the decision effect and the dissociation between decision and confidence judgments (decision BF_10_ = 16.51; decision vs confidence difference BF_10_ = 4.78). Figure 7 compares changes in categorical decisions and confidence judgments across all three experiments.

## General Discussion

Our data indicate that confidence can provide important diagnostic information for distinguishing sensory encoding aftereffects from aftereffects that do not change perception. Experiment 1 showed that an aftereffect driven by sensory adaptation^31–33^ can be measured equally well using categorical decisions and confidence judgments (see Figs 3–5). Experiment 2 showed that a non-perceptual decision bias (an instruction to make systematically biased decisions about ambiguous inputs) can dissociate categorisation biases from confidence reports; whereas categorical decisions provided evidence consistent with large aftereffects, confidence judgments suggested no change in sensory evidence (see Fig. 5). This implies that confidence can be informative about the cause of decision biases.

Having validated our approach in opposite contexts, Experiment 3 examined sequential direction decisions and associated confidence ratings about successive moving tests. Our results suggest that sensory adaptation can occur rapidly^13,14^: we observed an equal impact of the most recent trial on subsequent categorical decision and confidence judgments (see Fig. 6, left, and Fig. 7, 1-back results). These data mirror our results for an aftereffect known to be caused by sensory adaptation (Experiment 1), so these results suggest that the previous test can act as an adapting stimulus, generating a contrastive aftereffect that rapidly changes the perception of subsequent tests.

Overall, the present study implies that a participant’s insight into their own decision-making could resolve many contemporary debates in perception. Confidence reports leverage this insight to provide additional diagnostic information about the likely cause of a change in decision-making. When sensory adaptation produces a change to perception, this affects the evidence informing both categorical judgements and subjective confidence. In this case, an aftereffect should impact both measurements equally. However, when an aftereffect arises because people are making different decisions about ambiguous inputs, categorical decisions should dissociate from reported confidence. In that case, aftereffect measures selectively impact decision-making under uncertainty (i.e., low confidence). From the present evidence, we argue that this pattern of results is indicative of a non-perceptual aftereffect. Each of the three Experiments presented here supports this conclusion with independent and converging evidence.

Experiment 1 showed that, at baseline, spontaneous decision biases were separable from spontaneous confidence biases. This pattern of results suggests that random biases around the central tendency of the decision and confidence function can be independent. The effect of sensory adaptation on these response methods then provided converging estimates of perceptual changes, as measured by (1) the boundary between left- and right-directional categorisation, and (2) the peak uncertainty estimate measured from subjective confidence reports. In essence, Experiment 1 shows that sensory adaptation can be measured equally accurately, and with equal sensitivity, using either categorical decision-making or subjective confidence.

Experiment 2 used a visual cue to instruct participants to adopt a decision bias, and this bias dissociated the central tendency of decisions from the central tendency of confidence. These results are consistent with an earlier conceptually similar study, wherein participants were able to adopt a biased pattern of response in conditions of uncertainty, without having an adverse impact on measures of decisional precision (the slope of psychometric functions fit to decisional data, see ref.^26^). Our results replicate the key finding of this study–the ability of participants to adopt wilfully biased patterns of response, while also revealing a dissociation between the impact of this instruction on perceptual decisions and on measures of confidence.

We recognise that an instruction to adopt a systematic bias when people are uncertain could impact perceptual judgements differently compared to spontaneous decision biases. In which case, our approach might not distinguish between sensory coding changes and spontaneous decision biases. Equally plausible, however, is that decision biases will have an identifiable impact on categorisation regardless of whether the bias is instructed or spontaneous. Comparing the pattern of results across our Experiments, and by comparison between our study and other similar studies^26,38^, suggests that decision-level bias, regardless of origin, is identifiable by the dissociation of categorisation and confidence.

In Experiment 3 we found evidence for an internally generated bias, serial dependence^14,17,19^, which had an assimilative influence on sequential decision-making across trials. However, there was no evidence for a 2-back assimilative aftereffect for confidence judgments (see Fig. 6, right, and Fig. 7, 2-back results), which suggests that this categorisation bias was not based exclusively on sensory evidence^18,19,26^. We argue that this is likely driven by people being biased to repeat categorical decisions when inputs are ambiguous^28^ — the observed impact of prior stimuli is consistent with a post-perceptual influence of decision-making on perceptual judgements made under uncertainty. These results mirror the results of Experiment 2, where people were instructed to make systematically biased categorical decisions when inputs were ambiguous. The categorisation biases in this case, and in each of our three Experiments, were revealed by an unchanged region of subjective confidence. This pattern of dissociated decision and confidence judgements suggests that changes in decision processes can occur independently from changes in perceived sensory evidence. This effect was only apparent with the measure of confidence included in our experimental design.

Aftereffects estimated exclusively from categorical decisions only provide ambiguous evidence in favour of perceptual changes. Here we have shown that this evidence can be augmented with confidence measures. Using the classic motion-direction aftereffect (Experiment 1), we show that confidence is equally impacted by physiological processes that change perception. However, in each Experiment we also showed that categorisation judgements can change while confidence measures remain veridical — these data are otherwise suggestive of an ‘aftereffect’, but arose because people were making systematically biased judgments about perceptually ambiguous tests. Altogether, our data suggest that adding confidence judgments to categorical decision protocols (which are typically *exclusively* used to measure perceptual aftereffects) can reveal additional diagnostic information about the likely cause of a measured aftereffect.

The Experiment 3 data agree with many previous studies, showing that a motion aftereffect can be observed from brief exposures to motion signals^15–17^. Consistent with other researchers, we also found that brief exposure could produce both positive and negative motion aftereffects^17^ While it is interesting that decisions can be rapidly biased, we do not believe the time-course of a bias is diagnostic of its cause. In all cases where a perceptual decision must be reported, there is a possibility of a bias arising due to non-perceptual decision processes. This statement is equally true of biases resulting from long^31,32^ and short^15–17^ pre-exposure to moving inputs.

It is important to note that confidence was a highly sensitive measure for the aftereffects that arose because of physiological processes that change perception (see Fig. 7, compare Experiment 1, & Experiment 3: 1-back results). When dissociations between categorical decision and confidence measures arose (compare Experiment 1: bias split, Experiment 2a & 2b, & Experiment 3: 2-back results), it is unlikely that this could be attributed to confidence judgments having provided a poor, or insensitive, measure of a perceptual aftereffect.

The present approach is effective when the decision region of interest (the PSE) corresponds to the peak of directional uncertainty (where motion coherence is 0). A limitation, then, is that the relationship between the PSE and uncertainty is dependent on the shape (and statistical assumptions) of the respective decision and confidence distributions. The effectiveness of using confidence to diagnose perceptual and non-perceptual aftereffects might be limited when perceptual decisions are measured using other response methods (e.g., stasis judgements, where the decision distribution is a raised Gaussian function). In such cases, the relationship between decision-making and confidence might be more complex, or less diagnostic of perceptual aftereffects. We therefore encourage future experiments to examine this method in other domains, and for researchers to explore alternative ways in which perceptual and non-perceptual biases can be distinguished.

Judgements and decision-making are integral parts of reporting on perceptual experiences. Many studies have examined the effect of high-level cognition on perception^2–4,26,27,37,39,40^, and have observed robust changes in perceptual decisions. However, the extent to which aftereffects of this kind represent top-down changes to perception remains in dispute^1,3,12,18,26,41^. Expanding upon our results might help resolve current ambiguities about the causes of high-level aftereffects, and provide a means of directly testing for top-down effects of cognition on perception^1,3,5^. It remains a challenge, therefore, to discern the precise degree to which higher-order judgements affect sensory encoding. The present approach offers a simple method that could provide additional insight into these questions.

Other researchers, being mindful of the potential for non-perceptual factors to bias decision making, have proposed experimental protocols that minimise the influence of non-perceptual factors during perceptual decision making^38,42,43^. Another recent study used an approach similar to the confidence judgements used here. Participants made timed perceptual judgements, producing two data functions: a categorical function and a “chronometric curve”, similar in shape and assumptions to the psychometric function describing confidence in our study. Both the present study and the study by Witthoft and colleagues^44^ argue that sensory and decisional influences can combine to produce data consistent with an aftereffect, and that these effects can be parcelled by different tasks (here by confidence judgments, there by the speed of perceptual decisions).

Experiments examining sensory aftereffects have been vital to developing our understanding of the physiological processes that underlie perception and perceptual decision-making. Ultimately, however, we would like to be able to differentiate perceptual and post-perceptual processes, because people can make different decisions even when inputs might look, feel, or sound identical. Here we argue for a simple solution: use confidence judgements to identify the range of inputs that elicit greatest uncertainty as these will be disproportionately impacted by non-perceptual factors of judgement and decision-making. This can be achieved by asking participants to report on their confidence in each categorical perceptual decision.

## Conclusion

Confidence reports can provide additional information for determining if an aftereffect changes how the world looks, or sounds, or feels. Our approach could augment traditional protocols, at little cost in time and effort.

## Supplementary information

Supplementary item 1

## Data Availability

The datasets generated during and/or analysed during the current study are available from the corresponding author on reasonable request.
